# Neuroinflammation: Unraveling its role in neurodegenerative diseases

**DOI:** 10.1016/j.bas.2024.102866

**Published:** 2024-07-10

**Authors:** Muthu Thiruvengadam

**Affiliations:** a Department of Applied Bioscience, College of Life and Environmental Science, Konkuk University, Seoul, 05029, Republic of Korea; b Center for Global Health Research, Saveetha Medical College & Hospitals, Saveetha Institute of Medical and Technical Sciences (SIMATS), Thandalam, Chennai, 602 105, Tamil Nadu, India

This letter emphasizes the crucial role of neuroinflammation (NI) in the progression of neurodegenerative diseases and highlights its potential as a therapeutic target. By summarizing recent advancements in molecular biology and neuroimaging, this study sought to emphasize the dual nature of NI as both a response to neuronal damage and a driver of disease progression. It also aims to encourage further research to develop targeted anti-inflammatory treatments for Parkinson's disease (PD), Alzheimer's disease (AD), amyotrophic lateral sclerosis (ALS), and multiple sclerosis (MS). NI, an inflammation of nervous tissue, has emerged as a crucial player in the pathogenesis of various NDDs. Once considered as a secondary response to neuronal damage, NI has been identified as a primary contributor to disease progression in conditions such as PD, AD, ALS, and MS (Rahman et al., 2023). Recent advances in molecular biology and neuroimaging have provided deeper insights into the mechanisms driving NI and have highlighted potential therapeutic targets to mitigate its effects. At the cellular level, NI involves the activation of glial cells, including microglia and astrocytes, which are the main immune cells in the central nervous system (CNS). When microglia detect injury or pathogens, they shift from a resting to an activated state. This transition is marked by morphological changes and production of pro-inflammatory cytokines, including IL-1β, TNF-α, and IL-6 (Heneka et al., 2014). While acute activation of microglia is beneficial for clearing debris and pathogens, chronic activation leads to sustained inflammation, contributing to neuronal injury and disease progression (Qin et al., 2023). Astrocytes, another type of glial cells, significantly contribute to NI. When activated, astrocytes release various inflammatory mediators, such as cytokines, chemokines, and reactive oxygen species (ROS), intensifying neuronal damage and contributing to disease progression. Additionally, astrocytes undergo a process known as astrogliosis, which is marked by hypertrophy and enhanced expression of glial fibrillary acidic proteins. This procedure, while initially protective, can lead to scar formation and impede neuronal repair in chronic conditions (Sofroniew, 2015).

AD is characterized by the accumulation of Aβ plaques and neurofibrillary tangles composed of hyperphosphorylated tau proteins. NI has been increasingly identified as a critical component of AD pathology. Microglia are clustered around Aβ plaques, where they are activated and release inflammatory mediators that exacerbate plaque deposition and neuronal damage (Ansari et al., 2024). Recent studies have identified specific genetic risk factors, such as TREM2 variants, that influence microglial responses to Aβ, further linking NI to AD progression (Heppner et al., 2015). In PD, a decline in dopaminergic neurons in the substantia nigra is accompanied by notable neuroinflammatory reactions (Latif et al., 2021). Microglial activation and elevated levels of pro-inflammatory cytokines have been detected in the brains of patients with PD and animal models. Alpha-synuclein, a protein that aggregates in PD, directly activates microglia and triggers the release of inflammatory mediators that promote neuronal death. Understanding how alpha-synuclein aggregation and NI interact is essential for the development of therapeutic approaches targeting inflammatory responses in PD (Ransohoff, 2016). MS is an autoimmune condition characterized by loss of myelin sheaths around axons in the CNS. NI in MS involves complex interplay between the immune system and glial cells. Infiltrating T cells and macrophages interact with resident microglia and astrocytes, triggering the production of inflammatory cytokines and ROS, which damage myelin and axons. Recent progress in understanding of molecular mechanisms of MS, including the impact of the NLRP3 inflammasome on microglial activation, has revealed promising opportunities for therapeutic strategies (Freeman et al., 2017).

Targeting NI is a promising approach for NDD treatment. Several strategies are currently under investigation, including the use of anti-inflammatory drugs, immunomodulatory therapies, and agents that promote resolution of inflammation. For instance, nonsteroidal anti-inflammatory drugs have shown some efficacy in reducing the risk of AD, although their long-term use is limited by side effects (Heneka et al., 2014). More selective approaches, such as targeting specific cytokines or signaling pathways involved in NI, are being explored to minimize adverse effects and maximize therapeutic benefits (Heppner et al., 2015). In conclusion, NI plays a central role in the pathogenesis of NDDs by acting as both a response and a driver of neuronal damage. Progress in elucidating the molecular mechanisms underlying NI has underscored its potential as a target for therapeutic interventions. Ongoing research is crucial for unraveling the complexities of NI and for developing effective treatments that can arrest or slow the progression of these debilitating diseases.

## Disclosure statement

No potential conflict of interest was reported by author.

## Declaration of competing interest

The authors declare that they have no known competing financial interests or personal relationships that could have appeared to influence the work reported in this paper.
